# Does Pain Predict Frailty in Older Men and Women? Findings From the English Longitudinal Study of Ageing (ELSA)

**DOI:** 10.1093/gerona/glw226

**Published:** 2016-11-11

**Authors:** Katie F. Wade, Alan Marshall, Bram Vanhoutte, Frederick C. W. Wu, Terence W. O’Neill, David M. Lee

**Affiliations:** 1 Arthritis Research UK Centre for Epidemiology, Centre for Musculoskeletal Research, School of Biological Sciences, Manchester Academic Health Science Centre, The University of Manchester, UK.; 2 NIHR Manchester Musculoskeletal Biomedical Research Unit, Central Manchester University Hospitals NHS Foundation Trust, Manchester Academic Health Science Centre, UK.; 3 Department of Geography and Sustainable Development, School of Geography and Geosciences, University of St Andrews, UK.; 4 Cathie Marsh Institute for Social Research, School of Social Sciences, University of Manchester, UK.; 5 Andrology Research Unit, Developmental and Regenerative Biomedicine Research Group, Manchester Academic Health Science Centre (MAHSC), University of Manchester, UK.

**Keywords:** Frailty, Pain, Successful Ageing, ELSA

## Abstract

**Background:**

Pain has been suggested to act as a stressor during aging, potentially accelerating declines in health and functioning. Our objective was to examine the longitudinal association between self-reported pain and the development, or worsening, of frailty among older men and women.

**Methods:**

The study population consisted of 5,316 men and women living in private households in England, mean age 64.5 years, participating in the English Longitudinal Study of Ageing (ELSA). Data from Waves 2 and 6 of ELSA were used in this study with 8 years of follow-up. At Wave 2, participants were asked whether they were “often troubled with pain” and for those who reported yes, further information regarding the intensity of their pain (mild, moderate, or severe) was collected. Socioeconomic status (SES) was assessed using information about the current/most recent occupation and also net wealth. A frailty index (FI) was generated, with the presence of frailty defined as an FI >0.35. Among those *without* frailty at Wave 2, the association between pain at Wave 2 and frailty at Wave 6 was examined using logistic regression. We investigated whether pain predicted change in FI between Waves 2 and 6 using a negative binomial regression model. For both models adjustments were made for age, gender, lifestyle factors, depressive symptoms, and socioeconomic factors.

**Results:**

At Wave 2, 455 (19.7%) men and 856 (28.7%) women reported they often experienced moderate or severe pain. Of the 5,159 participants who were nonfrail at Wave 2, 328 (6.4%) were frail by Wave 6. The mean FI was 0.11 (standard deviation [*SD*] = 0.1) at Wave 2 and 0.15 (*SD* = 0.1) at Wave 6. After adjustment for age, gender, body mass index, lifestyle factors, and depressive symptoms, compared to participants reporting no pain at Wave 2 those reporting moderate (odds ratio [OR] = 3.08, 95% confidence interval [CI] = 2.28, 4.16) or severe pain (OR = 3.78, 95% CI = 2.51, 5.71) were significantly more likely to be frail at Wave 6. This association persisted after further adjustment for either occupational class and/or net wealth level. Compared to those without pain, those with mild, moderate, or severe pain were also more likely to develop worsening frailty, as assessed using the FI, and this association persisted after adjustment for SES. There was no evidence that the association between pain and frailty was influenced by gender.

**Conclusion:**

Pain is associated with an increased risk and intensity of frailty in older men and women. Socioeconomic factors contribute to the occurrence of frailty; though in our study do not explain the relationship between pain and frailty.

Frailty can be described as an increased vulnerability to stressors as it impairs multiple, inter-related systems, leads to decreases in physiological reserves and a decline in the ability to maintain homeostasis (1–3). Frailty is an important health problem through its association with adverse health outcomes including, disability, institutionalization, and death (1,4). The causes of frailty are complex and involve both biological and psychosocial factors (5,6).

Socioeconomic factors are an important determinant of frailty risk with evidence that lower socioeconomic status (SES) is associated with an increased risk of frailty (7–10). Marshall and colleagues (11) modeled cohort-specific trajectories in frailty among community-dwelling adults aged 50 years and older using five waves of the English Longitudinal Study of Ageing (ELSA), and showed that wealth differences impact on levels of frailty, with the fastest growth in frailty observed among the poorest participants. We reported recently an association between the occurrence of chronic widespread pain (CWP) and worsening frailty in a population sample of European men (12). The mechanism is unclear; pain experience may be linked with declining activity levels, or the presence of comorbidities which predispose to frailty. There is evidence that lower SES is also associated with CWP (13), chronic disabling pain (14) and disability due to pain (15). To our knowledge, however, there are no data examining whether the relationship between pain and frailty can be explained by SES.

Using data from ELSA, we examined the association between the occurrence and severity of pain and subsequent frailty development after 8 years of follow-up in older men and women. We hypothesized that pain occurrence and severity would be associated with the development and/or worsening of frailty. We also examined whether any observed associations could be explained by differences in sociodemographic factors.

## Methods

### Study Design

Details regarding study design have been described elsewhere (16). Briefly, men and women living in private households aged 50 years and over were recruited from the Health Survey of England cohort. The ELSA survey contains a broad range of information including demographic characteristics, mental and physical health, and social and economic circumstances collected from a combination of face-to-face interviews, nurse visits, and self-completed questionnaires. After the initial survey (conducted in 2002), participants were followed up and re-interviewed every 2 years. The current study uses data collected from the ELSA sample at Wave 2 (this being the first wave providing information on height and weight, collected between 2004 and 2005) and Wave 6 (2012–2013). At Wave 2, data were collected from 8,780 nationally representative participants and 5,316 participants provided data at both Waves 2 and 6, with 3,464 lost to follow-up over the 8-year period. Ethical approval was granted from the National Research and Ethics Committee.

### Assessments

#### Ascertainment of pain status

At Wave 2, participants were asked if they were “often troubled by pain”; if they responded “no,” their response was coded as “no pain” and for those who said yes, they were asked to evaluate the intensity of their pain on a 3-point scale (mild, moderate, severe). These two items were adopted from the Health and Retirement Survey and have been used in a previous publication assessing pain using ELSA (17). The pain variable was categorized as no pain, mild pain, moderate pain, or severe pain often.

#### Frailty

Frailty was defined in both Waves 2 and 6 datasets using an frailty index (FI) constructed of 51 deficits representing conditions that accumulate with age and are associated with adverse health outcomes (18) (Supplementary Table). The FI was adapted, from the FI created by Hubbard and colleagues (19), with 11 deficits being omitted; eight deficits were removed as they made up the Centre of Epidemiological Studies Depression (CES-D) scale, one referred to pain while walking and two were not repeated at Wave 6. The deficits used included functional and sensory impairments, self-report comorbidities, poor or fair self-rated health and a score in the lowest 10% of a composite measure of global cognitive function (20). Each subject’s deficit count was summed and divided by the total number of deficits considered. An FI was generated for each subject and was categorized according to published criteria (21); robust participants had FI scores <0.2, prefrail were 0.2–0.35 and frail were >0.35. For the purpose of the analysis, we defined incident frailty as those who were robust or prefrail at Wave 2 and who were identified as frail at Wave 6. The FI developed in ELSA has been used previously and validated as a predictor of mortality and institutionalization (22,23).

#### Socioeconomic status

Occupational class (current or most recent occupation) was measured using the National Statistics-Socio-Economic Classification scheme (NS-SEC), which provides a validated measure of a person’s social position determined using the nature of their employment contract (24). Net wealth was categorized in a previous publication as quintiles of the total net (nonpension) wealth measured at benefit unit level (benefit unit is a couple or single person with any dependent children). The wealth variable is estimated based on information regarding the value of all financial assets at the disposition of the benefit unit (ie, houses, businesses and savings) minus any debt.

#### Other assessments at Wave 2

Depressive symptoms were assessed using the CES-D scale. A score of four or more defines cases of elevated depressive symptoms (25). Weight (kg) and height (m) were assessed and body mass index (BMI) calculated as body weight (kg) divided by the square of height (m^2^). Physical activity was measured based upon the classification used in Allied Dunbar Survey of Fitness (26) and was categorized as; sedentary (sedentary occupation, engages in mild exercise 1–3 times a month or less), low (standing or sedentary occupation, engages in moderate exercise once a week or less or mild activity 1–3 times a month), moderate (physical work or engages in moderate activity more than once a week, or vigorous activity once a week/1–3 times a month) and high (heavy manual work or vigorous activity more than once a week). Participants were asked if they currently smoke cigarettes (at Wave 2) with responses recorded as yes or no.

#### Statistical methods

Descriptive statistics were used to summarize the sample characteristics. Appropriate parametric or nonparametric statistical approaches were used, to describe differences in Wave 2 characteristics between those who remained robust or prefrail at both time points and those who were robust or prefrail at Wave 2 and became frail by Wave 6. Logistic regression was used to examine the relationship between level of pain at Wave 2 and the new occurrence of frailty (Wave 6). To facilitate the longitudinal analysis only participants who were either robust or prefrail at Wave 2 were included in these models (ie, those with frailty [FI > 0.35] were excluded). The outcome was frailty status at Wave 6 (FI > 0.35 vs ≤ 0.35). Analyses were performed unadjusted, then adjusted for age and gender, further adjustment for BMI, smoking status, depressive symptoms (CES-D) and physical activity, then further adjustment for occupational class at Wave 2, or net wealth, or both occupation and net wealth. The results were expressed as odds ratios (OR) and 95% confidence intervals (CI). To examine change in FI over time, negative binomial regression analysis was performed, with pain at Wave 2 as the main predictor and FI at Wave 6 as the outcome, with adjustments made as outlined above in addition to FI at Wave 2. Results were reported as incident rate ratios (IRR) and 95% CI. Effect modification by gender was assessed by inclusion of interaction terms between pain and gender in the regression models. Analyses were conducted using STATA SE v13.1 (StataCorp, College Station, TX).

## Results

### Subject Characteristics

A total of 5,316 participants provided data for the analysis. Sample characteristics at Wave 2 are presented in Table 1. The mean age at Wave 2 was 64.5 years, and 56.3% were female.

**Table 1.** Wave 2 Participant Characteristics

Wave 2 Variable	All	Robust or Prefrail Both Time-Points	Robust/Prefrail at Wave 2, Frail at Wave 6	*P* ^†^
	Mean (SD), N* = 5,316	Mean (SD), N* = 4,831	Mean (SD), N* = 328	
Age (years)	64.5 (8.5)	63.9 (8.1)	72.5 (9.7)	≤.001
Body mass index (kg/m^2^)	26.9 (7.3)	26.9 (6.9)	26.3 (10.8)	.28
	**Number (%**)	**Number (%**)	**Number (%**)	
Sex				.29
Male	2,322 (43.7)	2,133 (44.2)	135 (41.2)	
Female	2,993 (56.3)	2,698 (55.8)	193 (58.8)	
Depression (CES-D)				≤.001
Not depressed	4,544 (86.7)	4,238 (88.7)	233 (73.3)	
Depressive symptoms	698 (13.3)	538 (11.3)	85 (26.7)	
Current smoker				.01
No	4,567 (86.2)	4,180 (86.7)	268 (82.0)	
Yes	734 (13.8)	641 (13.3)	59 (18.0)	
Physical activity				≤.001
High	1151 (21.8)	1125 (23.4)	22 (6.8)	
Moderate	2,830 (53.5)	2,672 (55.5)	132 (40.7)	
Low	1,146 (21.7)	942 (19.6)	130 (40.1)	
Sedentary	159 (3.0)	72 (1.5)	40 (12.3)	
Socio-economic classification (NS-SEC)				≤.001
Managerial and professional	1,797 (34.2)	1,685 (35.3)	82 (25.6)	
Intermediate	1,347 (25.7)	1,241 (26.0)	73 (22.7)	
Routine and manual	2,103 (40.1)	1,848 (38.7)	166 (51.7)	
Quintiles of net financial wealth				≤.001
1 = wealthiest	1,296 (24.7)	1,247 (26.2)	44 (13.6)	
2	1,173 (22.4)	1,107 (23.3)	45 (13.9)	
3	1,038 (19.8)	942 (19.8)	73 (22.6)	
4	810 (15.5)	696 (14.6)	70 (21.7)	
5 = poorest	918 (17.5)	765 (16.1)	91 (28.2)	
Pain status				≤.001
Severe pain	352 (6.6)	232 (4.8)	52 (16.0)	
Moderate pain	959 (18.1)	779 (16.2)	116 (35.8)	
Mild pain	579 (10.9)	540 (11.2)	32 (9.9)	
No pain	3,399 (64.3)	3,262 (67.8)	124 (38.3)	

Note: *Number of observations for each variable varies; 5,316 relates to complete age, BMI, and gender data.

^†^ *T*-test or chi-squared.

### Change in Frailty Status

Of those 5,159 participants who were nonfrail (robust or prefrail) at Wave 2, 328 (6.4%) had become frail by Wave 6 (41.2% male). The mean FI at Wave 2 was 0.11 (*SD* = 0.1) and at Wave 6, 0.15 (*SD* 0.1). The mean increase in FI between Waves 2 and 6 for participants nonfrail at Wave 2 was 0.04 (*SD* 0.09).

### Determinants of Incident Frailty

Among the 5,159 participants who were non-frail at Wave 2, compared to those who did not develop frailty by Wave 6, those who became frail were older (72.5 years vs 63.9 years), reported more depressive symptoms (26.7% vs 11.3%), were less physically active (6.8% vs 23.4% reported high levels of physical activity), were less likely to have managerial and professional occupations (25.6% vs 35.3%), were more likely to be in the poorest net wealth quartiles (28.2% vs 16.1%) and were more likely to report experiencing moderate (35.8% vs 16.2%) or severe pain (16.0% vs 4.8%) at Wave 2, Table 1.

### Pain Status and Incident Frailty

In participants who were nonfrail at Wave 2, compared to participants who reported no pain, after adjustment for age and gender, those who reported mild pain (OR = 1.65; 95% CI = 1.09, 2.49), moderate pain (OR = 3.81; 95% CI = 2.88, 5.04) and severe pain (OR = 5.68; 95% CI = 3.91, 8.24) at Wave 2, were more likely to have developed frailty at follow-up (Table 2, Model 1). After further adjustment for BMI, smoking, depressive symptoms and physical activity at Wave 2, those reporting moderate (OR = 3.08; 95% CI = 2.28, 4.16) and severe pain (OR = 3.78; 95% CI = 2.51, 5.71) were significantly more likely to have developed frailty at follow-up (Table 2, Model 2). Measures of SES were then added to the analysis; firstly an adjustment using occupation at Wave 2 was added to the model and compared to those who reported no pain, those experiencing moderate (OR = 3.01; 95% CI = 2.22, 4.09) and severe pain (OR = 3.72; 95% CI = 2.45, 5.64) were significantly more likely to have developed frailty at follow-up. When adjusting for the same previously identified risk factors but using net wealth as a marker of SES, compared to those who reported no pain at Wave 2, those with moderate pain (OR = 2.96; 95% CI = 2.18, 4.03) and those with severe pain (OR = 3.68, 95% CI = 2.42, 5.59) were significantly more likely to have developed frailty at follow-up. The results were broadly similar when both socioeconomic variables were included (Model 3). In Model 3, net wealth remained as an independent risk factor for frailty. There was evidence across all models of a dose response relationship between increasing severity of pain and frailty, that is, the higher the level of pain the greater the likelihood of developing frailty at Wave 6. The effect of any pain versus no pain on incident frailty was examined. After adjustments for age and gender, compared to those reporting no pain, those with any pain were significantly more likely to have developed frailty (OR = 3.38; 95% CI = 2.65, 4.31). Results remained significant after adjusting for previously identified risk factors and SES. There was no evidence that the association between pain and incident frailty differed by gender (*P*_interaction_ > 0.1).

**Table 2.** Wave 2 Pain Status and Incident Frailty: Logistic Regression Analysis

	Model 1, *N* = 5,137	Model 2, *N* = 4,724	Model 3, *N* = 4,601
Wave 2 Characteristics	Odds Ratio (95% Confidence Intervals)		
Age (years)	1.11 (1.10, 1.13)**	1.10 (1.09, 1.12)**	1.11 (1.09, 1.13)**
Gender (male vs female)	1.01 (0.80, 1.28)	0.79 (0.61, 1.03)	0.79 (0.60, 1.03)
Pain status			
No pain	Reference	Reference	Reference
Mild pain	1.65 (1.09, 2.49)*	1.34 (0.86, 2.11)	1.34 (0.84, 2.14)
Moderate pain	3.81 (2.88, 5.04)**	3.08 (2.28, 4.16)**	2.96 (2.17, 4.03)**
Severe pain	5.68 (3.91, 8.24)**	3.78 (2.51, 5.71)**	3.72 (2.44, 5.67)**
BMI (kg/m^2^)		1.00 (0.98, 1.01)	1.00 (0.98, 1.02)
Depression (CESD)			
Not depressed		Reference	Reference
Depressive symptoms		2.49 (1.84, 3.36)**	2.28 (1.67, 3.10)**
Smoking			
No		Reference	Reference
Yes		2.01 (1.42, 2.84)**	1.77 (1.24, 2.54)*
Physical activity			
High		Reference	Reference
Moderate		1.89 (1.17, 3.05)*	1.71 (1.06, 2.76)*
Low		3.93 (2.41, 6.42)**	3.31 (2.01, 5.43)**
Sedentary		11.06 (5.81, 21.05)**	9.54 (4.94, 18.42)**
Occupation			
Managerial and professional			Reference
Intermediate			0.84 (0.58, 1.23)
Routine and manual			1.17 (0.84, 1.62)
Net financial wealth quintiles			
1 = wealthiest			Reference
2			0.90 (0.56, 1.44)
3			1.53 (0.99, 2.36)
4			1.41 (0.89, 2.23)
5 = poorest			2.84 (1.82, 4.42)**

Note: In this analysis, individuals with frailty at baseline (Wave 2) were excluded. The outcome variable was frailty status (frailty index > 0.35 vs ≤ 0.35) at Wave 6. Model 1: adjusted for age and gender. Model 2: adjusted for age, gender, BMI, smoking status, depressive symptoms, and physical activity. Model 3: as Model 2 with additional adjustment for occupation and wealth.

*p ≤ .05. **p ≤ .001.

### Pain Status and Worsening Frailty

In all participants (including those who were frail at Wave 2), after adjusting for age, gender and the Wave 2 FI, compared to those with no pain, those who reported mild pain at Wave 2 had on average a 15% higher FI score at Wave 6 (Table 3, Model 1). Similarly, those who reported moderate pain had a 20% higher FI score at Wave 6 and those who reported severe pain had a 12% higher FI score at Wave 6 (Table 3, Model 1). After further adjusting for BMI, physical activity, smoking status, and depressive symptoms (Model 2) compared to those without pain those reporting mild pain, moderate pain or severe pain at Wave 2 had a 15%, 19%, and 9% higher FI score at Wave 6, respectively. The results were essentially unchanged following additional adjustments for SES (Model 3).

**Table 3.** Wave 2 Pain Status and Change in Frailty: Negative Binomial Regression Analysis

	Model 1, *N* = 5,289	Model 2, *N* = 4,859	Model 3, *N* = 4,733
Wave 2 Characteristics	Incident Rate Ratio (95% Confidence Intervals)		
Age (years)	1.03 (1.02, 1.03)**	1.03 (1.02, 1.03)**	1.03 (1.03, 1.03)**
Gender (male vs female)	1.00 (0.97, 1.03)	0.98 (0.95, 1.01)	0.97 (0.94, 1.00)
Pain status			
No pain	Reference	Reference	Reference
Mild pain	1.15 (1.09, 1.20)**	1.15 (1.09, 1.20)**	1.15 (1.10, 1.21)**
Moderate pain	1.20 (1.15, 1.26)**	1.19 (1.14, 1.25)**	1.19 (1.14, 1.24)**
Severe pain	1.12 (1.06, 1.20)**	1.09 (1.02, 1.17)*	1.10 (1.03, 1.17)*
BMI (kg/m^2^)		1.00 (1.00, 1.01)**	1.00 (1.00, 1.01)**
Depression (CESD)			
Not depressed		Reference	Reference
Depressive symptoms		1.07 (1.02, 1.12)*	1.05 (1.00, 1.10)*
Smoking			
No		Reference	Reference
Yes		1.17 (1.12, 1.23)**	1.12 (1.07, 1.17)**
Physical activity			
High		Reference	Reference
Moderate		1.14 (1.09, 1.18)**	1.13 (1.08, 1.17)**
Low		1.19 (1.14, 1.26)**	1.17 (1.12, 1.23)**
Sedentary		1.15 (1.05, 1.27)*	1.15 (1.04, 1.27)*
Occupation			
Managerial and professional			Reference
Intermediate			1.02 (0.98, 1.06)
Routine and manual			1.09 (1.05, 1.13)**
Quintiles of net financial wealth			
1 = wealthiest			Reference
2			1.03 (0.99, 1.08)
3			1.08 (1.03, 1.13)*
4			1.10 (1.05, 1.16)**
5 = poorest			1.17 (1.11, 1.24)**
Frailty index (FI)	1.05 (1.05, 1.05)**	1.05 (1.04, 1.05)**	1.04 (1.04, 1.05)**

Note: In this analysis, the outcome is frailty index (continuous variable). Model 1: adjusted for age, gender, and FI at Wave 2. Model 2: adjusted for age, gender, BMI, smoking status, depressive symptoms (CESD), physical activity, and FI at Wave 2. Model 3: as Model 2 with additional adjustments.

In Model 3, net wealth and occupation remained as independent risk factors for frailty. The effect of any pain versus no pain on worsening frailty was explored; in comparison to those with no pain at baseline, those with any pain had a 17% higher FI score at follow-up, after adjusting for age and gender; results remained significant after further adjustments for previously identified risk factors and SES. There was no evidence that the association between pain and change in FI differed by gender (*P*_interaction_ > 0.1).

## Discussion

This is the first study using data from a nationally representative survey to examine the prospective association between the occurrence of pain and the subsequent risk of developing frailty in both older men and women. Our findings agree with our previous observations in men that pain is predictive of incident and worsening frailty (12) and now extend these data to include women. The strength of the association between pain and the new occurrence of frailty increased with the severity of pain suggesting a dose–response relationship. Our data show an association between SES and frailty; participants in the poorest wealth quartile and those in manual and routine occupations were more likely to develop frailty or experience worsening frailty after 8 years of follow-up. Our results, however, suggest that SES did not explain the association between pain and subsequent development or worsening of frailty.

Previous studies have linked socioeconomic factors with pain. A cross-sectional study of 8,970 participants aged 40 years and older, showed that chronic pain was reported more often among manual workers than among managers (14). We found that SES and pain were risk factors for frailty, independently of one another. There are likely contextual factors that are linked to SES which increase the incidence of frailty. Our findings are in agreement with previous observational data linking SES with frailty. In a cross-sectional analysis of the Women’s Health and Aging Studies the odds of frailty (determined using the Fried phenotype) were increased for those with lower income and education irrespective of race (27). Using data collected from 4,000 people aged 65 years and older, Woo and colleagues found that socioeconomic position (as determined using education and income) both directly and indirectly (via lifestyle factors) affected frailty as measured using an FI (28).

What is the mechanism linking pain and frailty? It is possible that pain, whether chronic and/or severe, could potentially impact upon multiple physiological systems, thereby reducing reserve and in turn an individual’s ability to maintain homeostasis (29). Pain, and the adverse aspects of the pain experience may create a state of vulnerability to stressors which could explain why people with pain are at increased risk of developing, or experiencing worsening, frailty. This may provide a mechanistic interpretation for the evidence of a dose–response found between pain and frailty; the more pain a person suffers, potentially the greater the stress experienced and therefore the greater risk of frailty. Older adults experiencing pain are less physically active (30), experience more comorbidities (31) and worse functional mobility (32), than older adults without pain. These adverse consequences of pain may be responsible for the increase in risk of developing frailty.

We found that gender did not significantly influence the association between pain and frailty. This is an important observation as this is the first study, to our knowledge, that has explored the impact gender has on the longitudinal association between pain and frailty. We observed a dose–response relationship between increasing severity of pain and the onset of frailty. This was not seen, when we looked at the relationship between pain and worsening frailty, the reason for which is unclear. The results of the current study are also consistent with previous reports concerning the association between frailty and a variety of health and lifestyle factors. Mezuk and colleagues (33) reviewed 39 publications and found empirical evidence for a bidirectional association between frailty and depression in older adults, while Woods and colleagues (34) reported a strong relationship between depressive symptoms and incident frailty. We also found that low levels of physical activity are significantly associated with the development of frailty and this is consistent with data suggesting that exercise is associated with a reduced risk of frailty (as defined by gait speed and inability to rise from a chair without using one’s arms) (35).

Our study had a number of strengths. The prospective data were derived from a representative, population-based sample and included standardized methods and validated questionnaire instruments. Nonetheless, there are certain limitations that need to be considered. Of the 8,780 participants in Wave 2, 3,464 did not contribute data to Wave 6. Compared to those who took part at Wave 6 those who did not were older (70.6 years vs 64.5 years), more likely to be male (47% vs 44%) and to report “any” pain (42% vs 36%). Caution is needed in interpreting findings of the proportion of participants who changed frailty status or the mean change in FI between waves. Differences between participants who contributed data, and those who were lost to follow-up, are unlikely to have influenced results which were based on internal comparisons of participants who contributed data to both waves. Participants completed similar questionnaires during different study waves and it is unclear whether results were affected by this, as this can lead to participants changing their responses over time (36). Data collected on pain was obtained using self-report and errors of recall may have impacted on levels of pain reported, however, it is likely misclassification of pain would attenuate any observed associations. There was no specific time-frame linked with the pain question, but we feel asking whether participants were “often troubled by pain” would exclude those with acute pain though we cannot be certain. The study was prospective; caution is needed in interpreting the observed associations which do not provide evidence of causation. A potential weakness of the occupational measure, is that it does not capture participants who do not work and so we used net wealth as another measure. In a separate analysis, age at which participants left education was used as an alternative SES measure, and results were broadly similar (results not shown). The FI was modified from an index used in other studies. We excluded several deficits and this may have impacted on the psychometric properties of the tool. However, frailty indices are robust in terms of their performance, provided there are at least 30 deficits included in their derivation (18). The FI used contained a deficit regarding arthritis and although the presence of arthritis does not imply presence of pain, it is a common symptom of arthritis. We repeated our analysis after removing the arthritis deficit, and results were similar to the analysis when it was included (results not shown). Finally, the cohort was predominantly Caucasian, with a mean age of 64.5 years and caution is needed when extrapolating findings beyond this group.

In conclusion, self-reported pain was associated with both incident and worsening frailty in both older men and women, and this association was not explained by socioeconomic factors. Our findings highlight the importance of identifying pain symptoms in an older population and suggest potential opportunities for targeting individuals suffering from pain for interventions to reduce the occurrence of frailty. Further studies are needed to identify the physiological mechanisms underpinning this association and should aim to identify aspects of the pain experience responsible for the elevated risk for developing frailty.

## Supplementary Material

Supplementary material can be found at: http://biomedgerontology.oxfordjournals.org/

## Supplementary Material

Supplementary_Table
