# Supplementary material for: Does Pain Predict Frailty in Older Men and Women? Findings From the English Longitudinal Study of Ageing (ELSA)
Source: J Gerontol A Biol Sci Med Sci. 2016 Nov 11;72(3):403–9. doi: 10.1093/gerona/glw226 (PMC5861874; doi:10.1093/gerona/glw226)
Supplement: Supplementary_Table [file glw226_suppl_supplementary_table.docx]

**Supplementary Table 1** Deficit variables included in the ELSA-FI

| Description | Assigned values (1 indicates a deficit, 0 no deficit) | | | | | |
| --- | --- | --- | --- | --- | --- | --- |
| Difficulty with walking 100 yards | No=0 | Yes=1 |  |  |  |  |
| Difficulty sitting for about two hours | No=0 | Yes=1 |  |  |  |  |
| Difficulty getting up from a chair after sitting for long periods | No=0 | Yes=1 |  |  |  |  |
| Difficulty climbing several flights of stairs without resting | No=0 | Yes=1 |  |  |  |  |
| Difficulty climbing one flight of stairs without resting | No=0 | Yes=1 |  |  |  |  |
| Difficulty stooping, kneeling, or crouching | No=0 | Yes=1 |  |  |  |  |
| Difficulty reaching or extending arms above shoulder level | No=0 | Yes=1 |  |  |  |  |
| Difficulty pulling or pushing large objects like a living room chair | No=0 | Yes=1 |  |  |  |  |
| Difficulty lifting or carrying weights over 10 pounds, like a heavy bag | No=0 | Yes=1 |  |  |  |  |
| Difficulty picking up a 5p coin from a table | No=0 | Yes=1 |  |  |  |  |
| Difficulty dressing, including putting on shoes and socks | No=0 | Yes=1 |  |  |  |  |
| Difficulty walking across a room | No=0 | Yes=1 |  |  |  |  |
| Difficulty bathing or showering | No=0 | Yes=1 |  |  |  |  |
| Difficulty eating, such as cutting up your food | No=0 | Yes=1 |  |  |  |  |
| Difficulty getting in or out of bed | No=0 | Yes=1 |  |  |  |  |
| Difficulty using the toilet, including getting up or down | No=0 | Yes=1 |  |  |  |  |
| Difficulty using a map to figure out how to get around in a strange place | No=0 | Yes=1 |  |  |  |  |
| Difficulty preparing a hot meal | No=0 | Yes=1 |  |  |  |  |
| Difficulty shopping for groceries | No=0 | Yes=1 |  |  |  |  |
| Difficulty making telephone calls | No=0 | Yes=1 |  |  |  |  |
| Difficulty taking medications | No=0 | Yes=1 |  |  |  |  |
| Difficulty managing money, (e.g. paying bills and keeping track of expenses) | No=0 | Yes=1 |  |  |  |  |
| Difficulty doing work around the house or garden | No=0 | Yes=1 |  |  |  |  |
| Self-reported general health | No=0 | Yes=1 |  |  |  |  |
| High blood pressure or hypertension (self-reported) | No=0 | Yes=1 |  |  |  |  |
| Angina (self-reported) | No=0 | Yes=1 |  |  |  |  |
| Heart attack (including MI or coronary thrombosis) (self-reported) | No=0 | Yes=1 |  |  |  |  |
| Congestive heart failure (self-reported)  An abnormal heart rhythm (self-reported) | No=0  No=0 | Yes=1  Yes=1 |  |  |  |  |
| Diabetes or high blood sugar (self-reported) | No=0 | Yes=1 |  |  |  |  |
| A stroke (cerebral vascular disease) (self-reported) | No=0 | Yes=1 |  |  |  |  |
| Chronic lung disease such as chronic bronchitis or emphysema (self-reported) | No=0 | Yes=1 |  |  |  |  |
| Asthma (self-reported) | No=0 | Yes=1 |  |  |  |  |
| Arthritis (including osteoarthritis , or rheumatism) (self-reported) | No=0 | Yes=1 |  |  |  |  |
| Osteoporosis, sometimes called thin or brittle bones (self-reported) | No=0 | Yes=1 |  |  |  |  |
| Cancer or a malignant tumor (excluding minor skin cancers) (self-reported) | No=0 | Yes=1 |  |  |  |  |
| Parkinson's disease (self-reported) | No=0 | Yes=1 |  |  |  |  |
| Any emotional, nervous or psychiatric problems (self-reported) | No=0 | Yes=1 |  |  |  |  |
| Alzheimer's disease (self-reported) | No=0 | Yes=1 |  |  |  |  |
| Dementia, organic brain syndrome, senility or any other serious memory impairment (self-reported) | No=0 | Yes=1 |  |  |  |  |
| Self-reported eyesight (while using lenses, if appropriate) | Excellent=0 | V.good=0.2 | Good=0.4 | Fair=0.6 | Poor=0.8 | Blind=1 |
| Self-reported hearing (while using hearing aid if appropriate) | Excellent=0 | V.good=0.25 | Good=0.5 | Fair=0.75 | Poor=1 |  |
| Whether respondent has fallen down at all /last year /last 2years | No=0 | Yes=1 |  |  |  |  |
| Whether respondent has fractured hip ever /in last 2 years | No=0 | Yes=1 |  |  |  |  |
| Whether respondent has had joint replacement ever | No=0 | Yes=1 |  |  |  |  |
| Identify today's date: day of month | Yes=1 | No=0 |  |  |  |  |
| Identify today's date: month | Yes=1 | No=0 |  |  |  |  |
| Identify today's date: year | Yes=1 | No=0 |  |  |  |  |
| Identify the day of the week? | Yes=1 | No=0 |  |  |  |  |
| Immediate word recall (sample organized into quartiles) | 1st quintile=0 | 2nd quintile=0.3 | 3rd quintile=0.6 | 4th quintile=1 |  |  |
| Delayed word recall (sample organized into quintiles) | 1st quintile=0 | 2nd quintile=0.25 | 3rd quintile=0.5 | 4th quintile=0.75 | 5th quintile =1 | 6th quintile=1 |
